# Is Sublingual Immunotherapy the Final Answer? Implications for the Allergist

**DOI:** 10.1097/WOX.0b013e31816d92d6

**Published:** 2008-04-15

**Authors:** Giorgio Walter Canonica, Giovanni Passalacqua

**Affiliations:** 1 Allergy and Respiratory Diseases, Department of Internal Medicine, University of Genoa, Padiglione Maragliano, L.go R. Benzi 10, 16132 Genoa, Italy

**Keywords:** sublingual immunotherapy, safety, efficacy, clinical practice

## Abstract

Sublingual immunotherapy (SLIT) is now accepted as a viable alternative to the traditional injection route based on more than 40 clinical trials and several meta-analyses of efficacy. In addition, the safety profile is very favorable, also in younger children. Although some aspects need to be further clarified (eg, optimal doses, patient selection, and mechanisms of action), SLIT can be currently regarded as an additional therapeutic option that allergists have available. The main distinctive feature of SLIT is certainly its tolerability, safety, and convenience for the patient. Nonetheless, as happens with injection immunotherapy, it is mandatory that the prescription of SLIT is made by a trained specialist, and that a detailed diagnosis is made before prescribing it.

## General Aspects

Since its empirical discovery, [1] immunotherapy was given via subcutaneous injections (SCIT). Nevertheless, other modalities of administration were proposed and investigated during the 20th century. The rationale was either of desensitizing the target organs (nose or bronchi) or of achieving a systemic desensitization by administering the allergen orally. The sublingual administration was proposed at the beginning of the 1980s, and the first double-blind placebo-controlled (DBPC) study was published in 1986 [2]. Of note, this happened just after the British Committee for the Safety of Medicines had reported several deaths certainly caused by SCIT and had raised serious concerns about the safety and the risk-benefit ratio of the treatment [3]. An impressive number of clinical trials with sublingual immunotherapy (SLIT) have been published in less than 20 years, meta-analyses on the efficacy became available, and additional data on the safety, compliance, and mechanisms were also published. So far, SLIT is routinely used in many European countries, and very recently, clinical trials started also in the United States. In 1998, a panel of experts of the World Health Organization, based on an extensive review of the literature, concluded that SLIT is a viable alternative to SCIT, [4] and this statement was then confirmed in a position paper of the European Academy of Allergology and Clinical Immunology [5] and in the Allergic Rhinitis and its Impact on Asthma (ARIA) document [6] that extended the indications of SLIT also to children. Finally, a recent comprehensive review by the American Academy of Allergy Asthma and Immunology, despite a persisting skepticism on some points, acknowledged the clinical value of SLIT [7].

In Europe, SLIT is currently marketed by several manufacturers, and the administration schedules and the amount of allergen(s) vary depending on the producer, but the vaccines commercialized are standardized either biologically or immunologically. Recently, for many extracts, the content in micrograms of the major allergens has become available.

Sublingual immunotherapy is usually given as soluble tablets or drops to be kept under the tongue for 1 to 2 minutes and then swallowed. Based on clinical results and pharmaco-kinetic considerations, only the sublingual-swallow modality is considered the correct one, therefore, the acronym SLIT usually indicates the sublingual-swallow. Sublingual immunotherapy can be administered either preseasonally (stop at the beginning of the season), precoseasonally (stop at the end of the season), or continuously. Precoseasonal schedules are commonly the choice for pollen allergy, whereas for perennial allergens, continuous treatments are preferred. The amount of allergen given during a course of SLIT is usually higher than in an equivalent SCIT, therefore, the treatment has been also termed *high-dose SLIT*.

## Clinical Efficacy

At present, there are more than 40 randomized, double-blind, and placebo-controlled trials with SLIT (for review see Cox et al [7] and Passalacqua and Durham [8]). Most of the trials have confirmed the clinical efficacy of SLIT in allergic rhinitis caused by grasses, trees, ragweed, *Parietaria*, and mites. Only a few studies with mites [9,10] and grasses [11] failed to demonstrate a significant difference between active and placebo groups. In 2 recent large trials, the magnitude of the effect over placebo on symptoms and drug use was reported to be, respectively, 16% and 28%, [12] and 30% and 38% [13]. The largest study available so far, including more than 800 patients, a clear dose dependence of the clinical effect was also demonstrated [12]. A meta-analysis of 22 trials and 979 patients up to and including September 2002 concluded a significant efficacy of SLIT over placebo in allergic rhinitis [14]. Another meta-analysis of the treatment of allergic rhinitis with SLIT in pediatric patients (aged 4-18 years), involving 10 trials, showed that SLIT was effective as assessed by reductions in symptom scores and rescue medications [15]. The large majority of the studies was conducted in rhinitis, and in fact, the first meta-analysis stated that there were too few studies on the use of SLIT in allergic asthma to perform an evaluation. A meta-analysis in asthma was recently repeated, including 25 trials (either open or blinded) involving more than 1000 adults and children [16]. This meta-analysis demonstrated a significant effect of SLIT for most of the considered outcomes, including symptoms + medications, pulmonary function, and overall improvement.

It is likely that the good safety profile of SLIT would allow the expansion of its indications to conditions different from respiratory allergy. There is, for instance, 1 randomized controlled trial investigating the effects of SLIT in isolated allergic conjunctivitis caused by mites [17]. In this study, a significant clinical efficacy was demonstrated at the second year of therapy, and an increase in the conjunctival provocation threshold was also seen at the first year. One recent study [18]. demonstrated that SLIT is clinically effective in the treatment of immunoglobulin E-mediated food allergy to hazelnut, as testified by the increase in the threshold oral provocation dose. A recent randomized trial reported also a positive outcome in children with mild-to-moderate atopic dermatitis [19].

## Clinical Safety

The main rationale of SLIT is of minimizing the risks for adverse events; therefore, particular attention has been paid to safety in the published studies. The most frequently and commonly reported side effect is the onset of oral/sublingual itching after taking the dose. This phenomenon was always described as mild and self-resolving. Headache, rhinorrhea, constipation, and urticaria were reported only sporadically, and their incidence did not differ from the placebo groups. Noticeably, no fatal adverse event has been reported in the literature. The most recent review of the existing literature [7] reported a total occurrence of 14 severe adverse events (mainly asthma) in 20 years of clinical trials. So far, there are 3 reports of anaphylaxis probably caused by SLIT [20-22]. A controlled dose-finding study of safety [23] involved 48 grass-allergic patients outside the pollen season. They received SLIT for 28-day periods at progressively increasing doses, up to 200 μg Phl p 5 allergen that is about 40 times the amount given with 1 injection. The overall incidence of side effects was 74%, all of mild or moderate intensity. The most frequently reported events were irritation of the throat and oral itching.

Postmarketing surveys usually provide more realistic information on the safety in everyday clinical practice. There are now several postmarketing surveys conducted both in children and adults [24-27] available. Based on these large surveys, the overall rate of side effects ranged between 3% and 18% of patients and was invariantly less than 1 reaction per 1000 doses. More recently, it has been shown that the safety profile in children younger than 5 years is optimal as well [25].

## Practical Implications

The literature confirms the clinical efficacy and safety of SLIT. Thus, this modality of administration represents a new therapeutic tool for the allergist to use in clinical practice. The main clinical implication for physicians is of course the good safety profile that allows the patient to self-manage the therapy at home. In this regard, because SLIT is self-administered by patients themselves, concerns about the compliance have been raised. In recent years, some studies have attempted to quantify the adherence to therapy in the case of SLIT by means of unscheduled telephone interviews. This could be done as the treatments were prepared as tablets or single-dose vials, so that it was easy to count the remaining dose and to calculate the adherence. In 1 study, with 126 adult patients receiving SLIT in tablets, the compliance was reported greater than 90% over 1 year [28]. In another study of 442 patients, the compliance measured at 3 and 6 months was reportedly higher than 75% in 86% of the patients [29]. A similar study was conducted in a population of 71 children, [30] and the results did not substantially differ from adults. In fact, compliance data were available for all children at 3 months and for 56 children at 6 months. At 3 months, 85% of subjects had a compliance rate greater than 75% (69% of them adhered greater than 90%). At 6 months, 84% had a compliance rate greater than 75% (66% of them adhered greater than 90%).

Another practical implication, of interest for the allergist is that, because of the optimal safety profile, it seems that a slow updosing phase is not necessary. This approach, with a steady dosage since the beginning, would result in a treatment that is more patient-friendly and convenient to manage. Preliminary experiences with the no-updosing confirmed the feasibility of this administration, [31] and a randomized trial compared the safety of the traditional updosing regimen with the no-updosing [32] in 135 patients, with no difference in safety. In fact, the most recent large randomized trials were all performed with the no-updosing regimen, and their results in terms of safety, in addition to efficacy, were as favorable as those of the studies performed with the traditional route. Of note, soluble tablets are a convenient and easy to manage modality for giving the treatment. Tablets have the advantages of simple usage, of avoiding possible dosing errors, and their time of dissolution in the oral cavity can be exactly fixed. It is likely that soluble tablets will be in the future the most suitable way of administration.

Concerning the costs, it is true that the cumulative dose of allergen given via sublingual route is higher than in SCIT, and therefore the cost of the vaccine is higher as well. Nonetheless, a gross estimate shows that the cost of the extract is effectively balanced by the reduced need for medical and nursing time, so that the global cost of SLIT is even less than that of SCIT. A formal cost-benefit analysis by a pharmacoeconomic model [33] showed that SLIT for pollinosis in patients with rhinitis and/or asthma leads to a significant saving (in terms of direct and indirect costs). The same was seen in the pharmacoeconomic analyses performed in the large grass-tablet studies [34,35].

## Conclusions

The treatment of respiratory allergy is based on allergen avoidance, pharmacological treatment, and immunotherapy. Immunotherapy is an allergen-oriented immunomodulation that affects the immune response to allergens and whose action develops over long periods (months). Sublingual immunotherapy represents a significant advance because of safety and a good acceptance profile. Nonetheless, the self-administration itself requires careful instruction and a detailed follow-up of the patients. Its prescription must be made only by a specialist after a detailed diagnosis has been established, and the expected benefit-cost ratio has been carefully evaluated. The clinical efficacy of SLIT in both asthma and rhinitis is now supported by a large number of controlled trials and meta-analyses. Nonetheless, some clinical points still need to be developed: (*a*) the optimal dose of allergen is probably the most important aspect to be defined; (*b*) the optimal duration to achieve the maximum benefit and, possibly, to achieve a long-term benefit plus a preventive effect; (*c*) the long-lasting and preventive effects have been so far demonstrated in a single trial; and (*d*) the mechanisms of action are overall poorly known.

As per guidelines, SLIT is indicated in patients with rhinitis or asthma or both, with low adherence or previous severe adverse reactions to SCIT. Although no evidence of an increased risk, for prudential reasons and in analogy to SCIT, SLIT it is not recommended in patients with severe asthma. In addition, the cost-efficacy and clinical value of SLIT in mild intermittent rhinitis is a matter of discussion. Certainly, SLIT should not be considered as a last-choice treatment, but a complement to drug treatment. It is important to remember that SLIT must not be regarded as a substitute for subcutaneous immunotherapy, but rather as an additional choice or therapeutic tool.

## References

[B1] Noon L Prophylactic inoculation against hay fever Lancet 1911 1 1572 1573

[B2] Scadding K Brostoff J Low dose sublingual therapy in patients with allergic rhinitis due to dust mite Clin Allergy 1986 1 483 491 10.1111/j.1365-2222.1986.tb01983.x 3536171

[B3] Committee on the Safety of Medicines CSM update. Desensitizing vaccines Br Med J 1986 1 948

[B4] Bousquet J Lockey R Malling HJ World Health Organization Position Paper. Allergen immunotherapy: therapeutical vaccines for allergic diseases Allergy 1998 1 suppl 52 3 15 9802362 10.1016/s0091-6749(98)70271-4

[B5] Malling HJ EAACI position paper on local immunotherapy Allergy 1998 1 933 10.1111/j.1398-9995.1998.tb03793.x 9821472

[B6] Bousquet J Van Cauwenberge P Allergic rhinitis and its impact on asthma J Allergy Clin Immunol 2001 1 5 suppl S147 S334 11707753 10.1067/mai.2001.118891

[B7] Cox LS Linnemann DL Nolte H Weldon D Finegold I Nelson HS Sublingual immunotherapy: a comprehensive review J Allergy Clin Immunol 2006 1 1021 1035 10.1016/j.jaci.2006.02.040 16675328

[B8] Passalacqua G Durham SR ARIA update: allergen immunotherapy J Allergy Clin Immunol 2007 1 881 991 10.1016/j.jaci.2007.01.045 17418661

[B9] Hirsch T Sahn M Leupold W Double blind placebo controlled study of sublingual immunotherapy with house dust mite extracts in children Pediatr Allergy Immunol 1997 1 21 27 10.1111/j.1399-3038.1997.tb00138.x 9260215

[B10] Guez S Vatrinet C Fadel R André C House dust mite sublingual swallow immunotherapy in perennial rhinitis: a double blind placebo controlled study Allergy 2000 1 369 375 10.1034/j.1398-9995.2000.00413.x 10782522

[B11] Lima MT Wilson D Pitkin L Roberts A Nouri-Aria K Grass pollen sublingual immunotherapy for seasonal rhinoconjunctivitis: a randomized controlled trial Clin Exp Allergy 2002 1 507 514 10.1046/j.0954-7894.2002.01327.x 11972594

[B12] Durham SR Yang WH Pedersen MR Johansen N Rak S Sublingual immunotherapy with once-daily grass-allergen tablets: a randomised controlled trial in seasonal allergic rhinoconjunctivitis J Allergy Clin Immunol 2006 1 802 809 10.1016/j.jaci.2005.12.1358 16630937

[B13] Dahl R Kapp A Colombo G de Monchy JG Rak S Efficacy and safety of sublingual immunotherapy with grass allergen tablets for seasonal allergic rhinoconjunctivitis J Allergy Clin Immunol 2006 1 434 440 10.1016/j.jaci.2006.05.003 16890769

[B14] Wilson DR Torres L Durham SR Sublingual immunotherapy for allergic rhinitis Allergy 2005 1 3 8 10.1111/j.1398-9995.2005.00699.x 15575924

[B15] Penagos M Compalati E Tarantini F Baena-Cagnani R Huerta Lopez J Passalacqua G Canonica GW Efficacy of sublingual immunotherapy in the treatment of allergic rhinitis in children. Meta analysis of randomized controlled trials Ann Allergy Asthma Immunol 2006 1 141 148 10.1016/S1081-1206(10)60004-X 16937742

[B16] Calamita Z Saconato H Bronhara Pelà A Nagib Atallah A Efficacy of sublingual immunotherapy in asthma. Systematic review of randomized clinical trials Allergy 2006 1 1162 1172 10.1111/j.1398-9995.2006.01205.x 16942563

[B17] Mortemousque B Bertel F De Casamayor J Verin P Colin J House-dust mite sublingual-swallow immunotherapy in perennial conjunctivitis: a double-blind, placebo-controlled study Clin Exp Allergy 2003 1 464 469 10.1046/j.1365-2222.2003.01622.x 12680861

[B18] Cisteró Bahima A Sastre J Enrique E Fernández M Alonso R Tolerance and effects on skin reactivity to latex of sublingual rush immunotherapy with a latex extract J Investig Allergol Clin Immunol 2004 1 17 25 15160438

[B19] Pajno GB Caminiti L Vita D Barberio G Salzano G Sublingual immunotherapy in mite-sensitized children with atopic dermatitis: a randomized, double-blind, placebo-controlled study J Allergy Clin Immunol 2007 1 164 170 10.1016/j.jaci.2007.04.008 17543376

[B20] Dunsky E Goldstein MF Dvorin MJ Belecanech G Anaphylaxis due to sublingual immunotherapy Allergy 2006 1 1235 10.1111/j.1398-9995.2006.01137.x 16942576

[B21] Antico A Pagani M Crema A Anaphylaxis by latex sublingual immunotherapy Allergy 2006 1 1236 10.1111/j.1398-9995.2006.01155.x 16942577

[B22] Eifan AO Keles S Bahceciler NN Barlan IB Anaphylaxis to multiple pollen allergen sublingual immunotherapy Allergy 2007 1 567 568 10.1111/j.1398-9995.2006.01301.x 17313400

[B23] Kleine-Tebbe J Ribel M Herold DA Safety of a SQ-standardised grass allergen tablet for sublingual immunotherapy: a randomized, placebo-controlled trial Allergy 2006 1 181 184 10.1111/j.1398-9995.2006.00959.x 16409193

[B24] Di Rienzo V Pagani A Parmiani S Passalacqua G Canonica GW Post-marketing surveillance on the safety of sublingual swallow immunotherapy in children Allergy 1999 1 1110 1113 10.1034/j.1398-9995.1999.00267.x 10536891

[B25] Di Rienzo V Musarra A Sambugaro R Minelli M Pecora S Canonica GW Passalacqua G Post marketing survey on the safety of sublingual immunotherapy in children below the age of 5 years Clin Exp Allergy 2005 1 560 564 10.1111/j.1365-2222.2005.02219.x 15898975

[B26] Fiocchi A Pajno G La Grutta S Pezzuto F Incorvaia C Safety of SLI T in children aged 3 to 7 years Ann Allergy Asthma Immunol 2005 1 254 258 10.1016/S1081-1206(10)61222-7 16200816

[B27] Lombardi C Gargioni S Melchiorri A Tiri P Falagiani P Canonica GW Passalacqua G Safety of sublingual immunotherapy with monomeric allergoid in adults: multicenter post-marketing surveillance study Allergy 2001 1 989 992 10.1034/j.1398-9995.2001.00181.x 11576079

[B28] Lombardi C Gani F Landi M Falagiani P Bruno M Canonica GW Passalacqua G Quantitative assessment of the adherence to sublingual immunotherapy J Allergy Clin Immunol 2004 1 1219 1220 10.1016/j.jaci.2004.03.013 15214362

[B29] Passalacqua G Musarra A Pecora S Amoroso S Antonicelli L Quantitative assessment of the compliance with a once daily sublingual immunotherapy in real life J Allergy Clin Immunol 2006 1 946 948 10.1016/j.jaci.2005.12.1312 16630956

[B30] Passalacqua G Musarra A Pecora S Amoroso S Antonicelli L Quantitative assessment of the compliance with once daily sublingual immunotherapy in children Pediatr Allergy Immunol 2007 1 58 62 10.1111/j.1399-3038.2006.00471.x 17295800

[B31] Guerra L Compalati E Rogkakou A Pecora S Passalacqua G Canonica GW Randomized open comparison of the safety of SLIT in a no updosing and traditional updosing schedule in patients with parietaria allergy Allergol Immunopathol 2006 1 82 83 10.1016/S0301-0546(06)73518-2 16808019

[B32] Rodriguez F Boquete M Ibanez MD de la Torre-Martinez F Tabar AI Once daily sublingual immunotherapy without updosing--a new treatment schedule Int Arch Allergy Immunol 2006 1 321 326 10.1159/000093710 16741368

[B33] Berto P Passalacqua G Ortolani C Senna G Crimi N Frati F Canonica GW Economic evaluation of sublingual immunotherapy versus symptomatic treatment in adults with pollen-induced respiratory allergy: the SPAI study Ann Allergy Asthma Immunol 2006 1 615 621 10.1016/S1081-1206(10)61090-3 17165269

[B34] Bachert C Vestenbaek U Christensen J Griffiths UK Poulsen PB Cost-effectiveness of grass allergen tablet (GRAZAX) for the prevention of seasonal grass pollen induced rhinoconjunctivitis--a Northern European perspective Clin Exp Allergy 2007 1 772 779 10.1111/j.1365-2222.2007.02706.x 17456225

[B35] Canonica GW Poulsen PB Vestenbaek U Cost-effectiveness of GRAZAX for prevention of grass pollen induced rhinoconjunctivitis in Southern Europe Respir Med 2007 1885 1894 17611095 10.1016/j.rmed.2007.05.003

